# Development of a Novel Electrochemical Biosensor Based on Carbon Nanofibers–Cobalt Phthalocyanine–Laccase for the Detection of p-Coumaric Acid in Phytoproducts

**DOI:** 10.3390/ijms22179302

**Published:** 2021-08-27

**Authors:** Alexandra Virginia Bounegru, Constantin Apetrei

**Affiliations:** Department of Chemistry, Physics and Environment, Faculty of Sciences and Environment, “Dunărea de Jos” University of Galaţi, 47 Domnească Street, 800008 Galaţi, Romania; alexandra.meresescu@ugal.ro

**Keywords:** p-coumaric acid, biosensor, laccase, cobalt phtalocyanine

## Abstract

The present paper developed a new enzymatic biosensor whose support is a screen-printed electrode based on carbon nanofibers modified with cobalt phthalocyanine and laccase (CNF-CoPc-Lac/SPE) to determine the p-coumaric acid (PCA) content by cyclic voltammetry and square wave voltammetry. Sensor modification was achieved by the casting and cross-linking technique, using glutaraldehyde as a reticulation agent. The biosensor’s response showed the PCA redox processes in a very stable and sensitive manner. The calibration curve was developed for the concentration range of p-coumaric acid of 0.1–202.5 μM, using cyclic voltammetry and chronoamperometry. The biosensor yielded optimal results for the linearity range 0.4–6.4 μM and stood out by low LOD and LOQ values, i.e., 4.83 × 10^−7^ M and 1.61 × 10^−^^6^ M, respectively. PCA was successfully determined in three phytoproducts of complex composition. The results obtained by the voltammetric method were compared to the ones obtained by the FTIR method. The amount of p-coumaric acid determined by means of CNF-CoPc-Lac/SPE was close to the one obtained by the standard spectrometric method.

## 1. Introduction

Coumaric acids are derivatives of the cinnamic acid that are mono-hydroxylated at the phenyl group, and the p-coumaric acid (PCA) is the most abundant isomer, where the OH group is in the *para* position to the side chain [1,2].

PCA is biosynthesized mainly from tyrosine in the case of microorganisms. As a lignine component, PCA is omnipresent in plants in a low concentration [3]. In plants, the biosynthesis of p-coumaric acid involves two chemical processes: the phenylalanine ammonium-lyase (PAL) first catalyzes the conversion of phenylalanine into trans-cinnamic acid, which is subsequently hydroxylated in the *para* position under the action of the trans-cinnamic 4-hydroxylaze enzyme (C4H) [4]. On the other hand, certain PAL enzymes may use tyrosine as an initial substrate (PAL/TAL), thus leading to the direct formation of p-coumaric acid from tyrosine, without the intermediary stage of the trans-cinnamic acid. In addition, there are certain specific ammonium-lyase enzymes in desaminating tyrosine (TAL 1) [5]. By introducing PAL/TAL or TAL 1, p-coumaric acid may be produced on the tyrosine pathway from microorganisms such as *Escherichia coli* [6], *Saccharomyces cerevisiae* [7], *Streptomyces lividans* [8], and *Pseudomonas putida* [9].

PCA has antioxidant [10,11,12], antibacterial [13,14,15], anti-inflammatory properties [16,17,18] and serves as a conventional precursor in manufacturing flavors and perfumes used in chemical products or foodstuffs. This compound has a wide range of applications in the nutraceutical, pharmaceutical, material, and chemical industries. Similarly, PCA serves as a raw material for producing biodegradable thermoplastic materials [19].

It has recently been discovered that the p-coumaric acid also has an antiproliferative [20], anxiolytic [21], neuroprotective [22], nephroprotective [20], hepatoprotective [23] effect, and it may inhibit melanogenesis [24]. In addition, PCA, as a metabolite on the phenylpropanoid pathway in plants, is a common precursor to the biosynthesis of numerous derivatives, such as other phenylpropanoidic compounds [25], flavonoids [26], stilbenes [27], and antocyans [28].

Taking into account the biological importance of p-coumaric acid, its detection in various food or pharmaceutical products becomes a necessity. In time, various analytical methods have been applied to analyze this hydroxycinnamic acid, such as capillary electrophoresis (CZE) [29], micellar electrokinetic chromatography (MEKC) with UV-detection [30], surface-enhanced Raman spectroscopy (SERS) [31], and high-performance liquid chromatography (HPLC) [32].

Voltammetric techniques, such as cyclic voltammetry, differential pulse voltammetry, or square wave voltammetry, have been commonly used to investigate the electrocatalytic activity and the electrooxidation mechanism of several analytes [33,34,35], including p-coumaric acid. These experimental studies used various electrodes such as the one made of Pt [36], glassy carbon electrode modified with multilayer carbon nanotubes [37], a modified sensor using a functionalized zeolite with magnetic nanoparticles of ferroferric oxide, Fe_3_O_4_@ ZIF4 [38], or a lab-on-a-chip type device with eight electrodes printed on a plastic substrate [39].

Developing an electrochemical biosensor may be a good fit for PCA detection, thus taking advantage of the recognition properties of an enzyme immobilized on an electrode surface. An enzyme active toward the ortho and para-diphenol groups, including mono-, di- and polyphenols, aminophenols, or methoxyphenols, is laccase (Lac) [40]. Lac was used in biosensors based on metallic nanoparticles [41], carbon nanomaterials [42], polymers, and various membranes such as Nafion [43] and chitosan [44]. Great importance is placed on the synergic combination of nanomaterials and laccase, which enhances the performance of electrochemical biosensors [45], being definitely useful in PCA detection. Using mediators with electrocatalytic properties, such as for instance metalphtalocyanines, may increase the speed of electron transfer and decrease the detection potential of analytes [46].

The purpose of this paper is to assess the electrochemical behavior of a new biosensor based on carbon nanofibers, cobalt phthalocyanine, and laccase (CNF-CoPc-Lac-SPE) in PCA detection by means of various voltammetric techniques. In addition, the electroanalytical method will be validated by the FTIR spectrometric method to quantify PCA in various phytoproducts.

## 2. Results and Discussion

### 2.1. Preliminary Studies for Electrode Characterization

**Figure 1:**
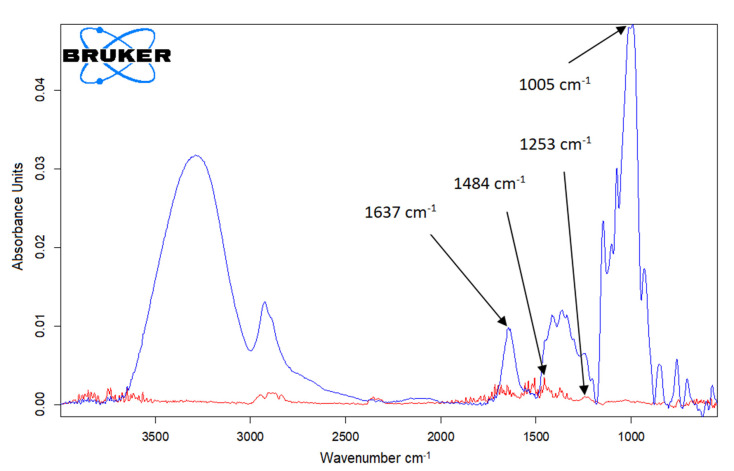
FTIR spectra for CNF-CoPc/SPE (red line) and CNF-CoPc-Lac/SPE (blue line).

To observe the changes in the sensor based on carbon nanofibers after modification with cobalt phyhalocyanine and laccase, the active surface of the two working electrodes was analyzed by the FTIR spectrotometric method, and the results are presented in Figure 1.

Figure 1 shows the FTIR spectra for CNF-Co-Pc/SPE and CNF-CoPc-Lac/SPE, respectively, showing the difference in the peak number and background noise.

The FTIR spectrum of CNF-CoPc/SPE compared to CNF-CoPc-Lac/SPE showed that there are clear differences in the wavelength range of 3000–500 cm^−1^ as can be seen in Figure 1 (red line).

In the case of FTIR analysis of CNF-CoPc/SPE, several sharp peaks are observed in the range at 4000–3500 cm^−1^ and 2000–500 cm^−1^. The CoPc spectrum showed a characteristic band at 731 cm^−1^, which corresponds to the Co-N vibration. The band at 1253 cm^−1^ due to the stretching vibrations C-N and the band at 1484 cm^−1^ can be attributed to the skeletal stretching vibration of the benzene ring C-C [47].

In Figure 1 (blue line) it can be seen that the FTIR spectrum has changed considerably. The peak at 1637 cm^−1^ could be attributed to the secondary amide bond (C=N) that occurs after cross-linking the laccase with glutaraldehyde [48,49], and the intense peak at 1005 cm^−1^ is related to the Cu=N stretching vibration in laccase.

The preliminary tests assessed the electrochemical behavior of CNF/SPE, CNF-CoPc/SPE, and CNF-CoPc-Lac/SPE in 0.1 M phosphate buffer solutions (PBS) with various pHs (3.0, 4.0, 5.0, 6.0). According to previous studies, the stable signal was obtained in the potential range −0.4 and +1.3 V [50]. As a result, this potential range was used to study the electrochemical behavior of the electrodes immersed in 0.1 M PBS (pH = 3.0, 4.0, 5.0, 6.0) at a scan rate of 0.1 V/s.

The cyclic voltammograms obtained for CNF/SPE did not display peaks within the potential range under study (the results are not presented), which proves that the active surface of the electrode does not exhibit contaminations and the carbon nanofibers are of high purity.

When the CNF-CoPc/SPE sensor is immersed in a phosphate buffer solution at various pH values, the cyclic voltammogram showed two peaks: an anodic one of low intensity and a cathodic one that is more obvious. The current of the cathodic peak increased with the pH. At pH = 5.0, the anodic peak occurs at 0.67 V, and the cathodic peak occurs at −0.21 V. The peaks are related to the oxido-reducing process of CoPc on the surface of the modified electrode, and they are in agreement with the results obtained in other studies [51].

**Figure 2:**
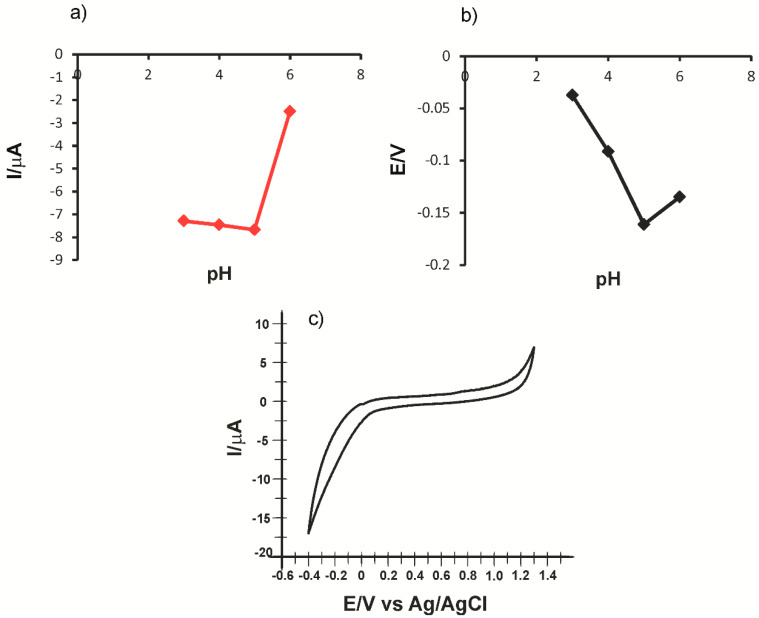
pH influence on the cathodic peak current intensity (**a**) and the cathodic peak potential (**b**) obtained by immersing CNF-CoPc-Lac/SPE in PBS and CV of CNF-CoPc-Lac/SPE immersed in PBS 0.1 M pH = 5.0 (**c**) CV parameters: scan rate 0.1 V/s and potential range from −0.4 to +1.3 V.

Previously, the electrochemical reduction of laccase was investigated on the biosensor surface in PBS solution at pH values ranging between 3.0 and 6.0, and the CV responses showed that I_pc_ increases in direct proportion with the pH increase, up to pH = 5.0. At pH = 6.0, I_pc_ decreased dramatically. Moreover, the increase in the pH up to 5.0 triggered a linear displacement of the cathodic peak potential to more negative values. The regression equation is E_p_ = −0.0362 pH + 0.0567. Figure 2 shows the influence of the pH on the laccase reduction process on the electrode surface (Figure 2a,b) and the CV aspect of CNF-CoPc-Lac/SPE immersed in PBS 0.1 M, pH = 5.0 (Figure 2c).

This electrochemical behavior shows that laccase activity is optimal at a rather acidic pH, which is confirmed in other studies [42,52]. Observing this, we established that the optimal pH would be at 5.0. In these experiments, we confirmed that it was at this pH that laccase activity was not adversely affected, and the enzyme immobilization was performed properly. At pH = 6.0, a current decrease occurs, which is probably due to loss or inactivation of the enzyme activity.

**Figure 3:**
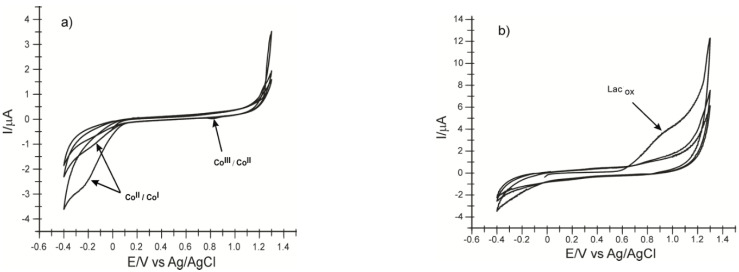
Cyclic voltammograms of CNF-CoPc/SPE (**a**) and CNF-CoPc-Lac/SPE (**b**) immersed in PBS solution 0.1 M pH = 5.0. Three successive cycles.

The cyclic voltammograms of the two modified electrodes immersed in 0.1 M PBS solution pH = 5.0 are shown in Figure 3. The aspect of the recorded CV shows the change of the electrodes. The signal was stabilized after three cycles.

The Co^II^/Co^I^ peak occurs at −0.239 V and varies depending on the scan cycles. This indicates that CoPc undergoes an adsorption process on the electrode surface. Another irreversible bit appears at approximately 0.821 V and can be attributed to the Co^III^/Co^II^ transition. In basic or acidic solutions, this process is reversible but usually with a lower intensity than the corresponding Co^II^/Co^I^ [53]. In this case, pH 5.0 does not favor the reversibility of the process. Figure 3b shows the oxidation of the laccase at the first scan at 0.923 V.

**Figure 4:**
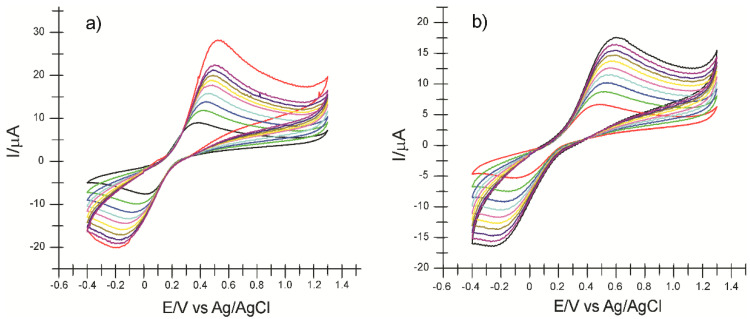
Cyclic voltammograms of CNF-CoPc/SPE (**a**), CNF-CoPc-Lac/SPE (**b**) immersed in 10^−^^3^ M K_4_[Fe(CN)_6_]—0.1 M KCl solution registered with scan rates in the range 0.1–1.0 V/s. The cyclic voltammograms with different colors correspond to different scan rates.

In the next step, cyclic voltammograms were recorded at different scan rates (0.1–1.0 V/s), using the 10^−3^ M–0.1 M PBS potassium ferrocyanide solution (pH 5.0). In Figure 4a,b, it can be seen that the intensity of the peaks corresponding to the oxidation-reduction processes of ferrocyanide increases with the increase in the scan rate. For all three electrodes, there is a linear dependence between the anodic peak current and the square root of the scan rate, which demonstrates that the electrochemical process is controlled by the diffusion of electroactive species [54]. The Randles-Sevcik equation was used to calculate the active area of the electrodes [55].

Using the linear regression equation I_pa_ vs. v ^1/2^ and the diffusion coefficient of the ferrocyanide ion D = 7.26 × 10^−6^ cm^2^/s, [56] the value of the area of the active surface for the three electrodes was calculated. The CNF-CoPc-Lac/SPE biosensor has the lowest value of the active surface (0.341 cm^2^), comparing with CNF/SPE (0.917 cm^2^) and CNF-CoPc/SPE (0.977 cm^2^), because the immobilized enzyme on the electrode surface does not participate in the process of oxidation-reduction of ferrocyanide, demonstrating selectivity. The two screen-printed sensors have a close active surface in value, but the modification of the electrode surface with cobalt phthalocyanine explains the larger active area of CNF-CoPc/SPE. Cobalt phthalocyanine was used as an electrons mediator, which facilitates the transfer of electrons, having a good biocompatibility for the enzymatic modification of the electrode [51]. Laccase ensures the selectivity of the detection of phenolic compounds [57,58].

### 2.2. The Voltammetric Responses of Electrodes in p-Coumaric Acid Solution

The catalytic activity of laccase ranges between a strongly acid environment and a slightly basic one, so that pH optimization is a key factor for biosensitivity [59]. Similarly, pH modifications affect the protonation mechanism involved in the electrochemical redox reaction of phenolic compounds [60].

According to preliminary studies, as well as the literature, it was found that the optimal pH value for phenolic compound detection is 5.0 [49]. The peaks obtained for this pH value are more obvious and clearly defined [59,61,62]. A greater peak intensity shows that the immobilization stage did not affect adversely the enzyme activity. A lower pH value may contribute to the faster degradation of the enzyme. Therefore, the following experimental tests used as support electrolyte the 0.1 M PBS at pH = 5.0.

The working electrodes were used to record the cyclic voltammograms, using a solution of p-coumaric acid 10^−3^ M (PBS 0.1 M pH = 5.0). The scan rate was 0.1 V/s. The cyclic voltammograms are slightly different according to the modifications of the working electrode. In each case, the first voltammetric scan shows a well-defined irreversible anodic peak, which is associated with the oxidation of the hydroxyl group on the aromatic ring of the molecule, and the formation of the phenoxy radicals, that may later dimerize or polymerize [37]. In the case of the biosensor, the anodic peak occurs at a potential of 0.904 V.

Upon successive scanning, the oxidation product of the p-coumaric acid is deposited on the electrode surface, forming a polymeric film, thus accounting for the occurrence of another reversible oxidation peak, at a lower potential than the one for p-coumaric acid [63].

The increased intensity in the reversible oxidation peak is explainable by the increase in the thickness of the polymeric film coating the electrode surface, which prevents the diffusion of the p-coumaric acid and its oxidation on the electrode surface [63]. The appearance of the anodic peak of the oxidation product, with a lower oxidation potential than that of the raw material, in this case p-coumaric acid, is due to the polymer formed by the oxidation of the respective monomer. The oxidized p-coumaric acid favors the additional deposition of the polymeric film, which led to the passivation of the electrode surface, thus favoring the redox process of the oxidation product.

**Figure 5:**
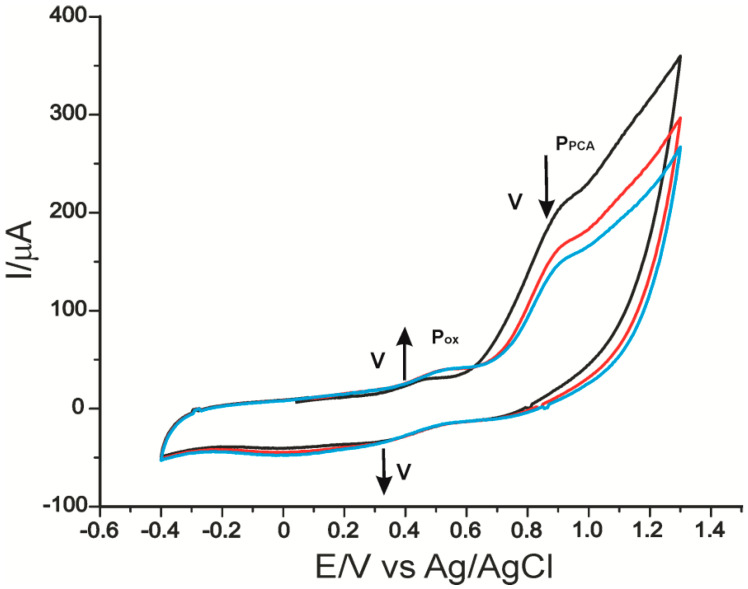
Cyclic voltammograms of CNF-CoPc-Lac/SPE immersed in p-coumaric acid solution 10^−3^ M (PBS 0.1 M electrolyte, pH = 5.0): first scan (black line), second scan (red line), and third scan (blue line).

When using CNF-CoPc-Lac/SPE, the maximum potential related to the oxidation product occurs at E_pa_ = 0.537 V, and the potential of the reduction peak is E_pc_ = 0.011 V. Thus, the quasi-reversibility of the oxidation process is confirmed. These values are similar to the ones found in other studies on the oxido-reduction process of the p-coumaric acid [63]. When the oxidation product is adsorbed on the electrode surface, the intensity of the oxidation peak of p-coumaric acid decreases, while the intensity of the peak associated to the oxidation product increases at successive scanning (Figure 5).

The peak related to the oxidation product has a lower oxidation potential than the one of p-coumaric acid, which is due to the formation of organic polymers through oxidation [63]. However, the sensor’s signals become stable after three cycles. The electrochemical behavior of coumaric acid is similar for all the working electrodes used, and the electrochemical parameters obtained are shown in Table 1.

CNF-CoPc-Lac/SPE stands out by a low value of the cathodic peak potential, which means that the reduction process requires a lower activation energy and is influenced by the presence of laccase [35,53,64,65]. Similarly, the low value of E_pc_ suggests a fast electron transfer process in the PCA redox process at the level of the active surface of the biosensor [66]. It can be seen that E_pa_^1^ related to the oxidation of p-coumaric acid at the CNF-CoPC-LaC/SPE is higher than the CNF/SPE and CNF-CoPC/SPE, while the cathodic peak potential is lower. We consider that this difference is due to the presence of laccase on the surface of the electrode which mainly catalyzes the reduction of the electroactive species in this situation.

As a result, CNF-CoPc-Lac/SPE exhibits better selectivity in comparison with the other two sensors in detecting p-coumaric acid, thus confirming the biocatalytic activity of the laccase immobilized on the biosensor surface. The values of the parameters E^1/2^ and I_pc_/I_pa_ prove that the biosensor is more sensitive.

In addition, the cathodic peak is visibly more intense, which is why subsequent calculations will be related to its modifications. In the case of CNF-CoPc-Lac/SPE, the signal was more stable, and the background noise was lower.

**Figure 6:**
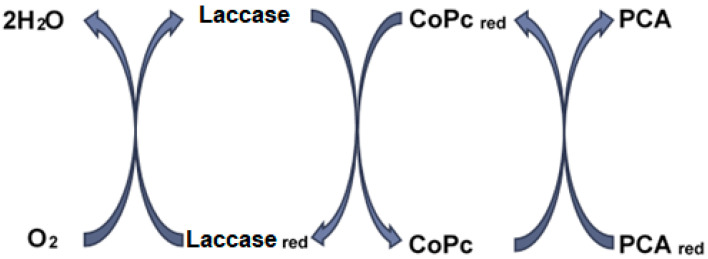
Scheme of p-coumaric redox process, adapted from [67,68].

Figure 6 shows the PCA oxidation mechanism by laccase through the mediation of cobalt phthalocyanine.

The reduction process of p-coumaric acid was studied, and it was shown that it occurs at low potential and is due to the pre-protonated conjugated double bond [69].

Taking into account that the p-coumaric acid molecule contains an oxidizing phenol group on the aromatic cycle, it may be presumed that this compound may be determined by voltammetry. The absence of the hydroxyl substitutes in the ortho position in the chemical structure of p-coumaric acid results in the irreversibility of the electrochemical redox processes, since the semiquinonic radical is not stabilized [70].

The mechanism of the biocatalytic action of laccase toward various substrates is related to the active center of the enzyme, containing four differently coordinated copper ions, leading the electrons from a reducing substrate to the molecular oxygen [71]. The first step is reducing the T1 copper sites by accepting an electron from the oxidized substrate. Then, the electron in the reduced T1 sites is transferred to the T2/T3 trinuclear group, together with the water generated by reducing the molecular oxygen [72]. Phenolic compounds may be directly oxidized by laccase and may be detected by a laccase-based biosensor. The oxidation reaction of laccase-catalyzed polyphenols is the following:

$${\mathrm{PH}}_{2}+\frac{1}{2}O_{2}\to P+H_{2}O$$

where P and PH_2_ are oxidized, and reduced respectively, phenolic compounds [73].

As a result, laccase is able to catalyze the oxidation process of p-coumaric acid, which is proved by the value close to 1 of the I_pc_/I_pa_ ratio for CNF-CoPc-Lac/SPE.

The peaks obtained for the scan rate 0.1 V/s have low intensity and are less visible due to the influence of the capacitive current. At higher scan rates, the Faradaic currents are higher, and the peaks are better defined [74].

**Figure 7:**
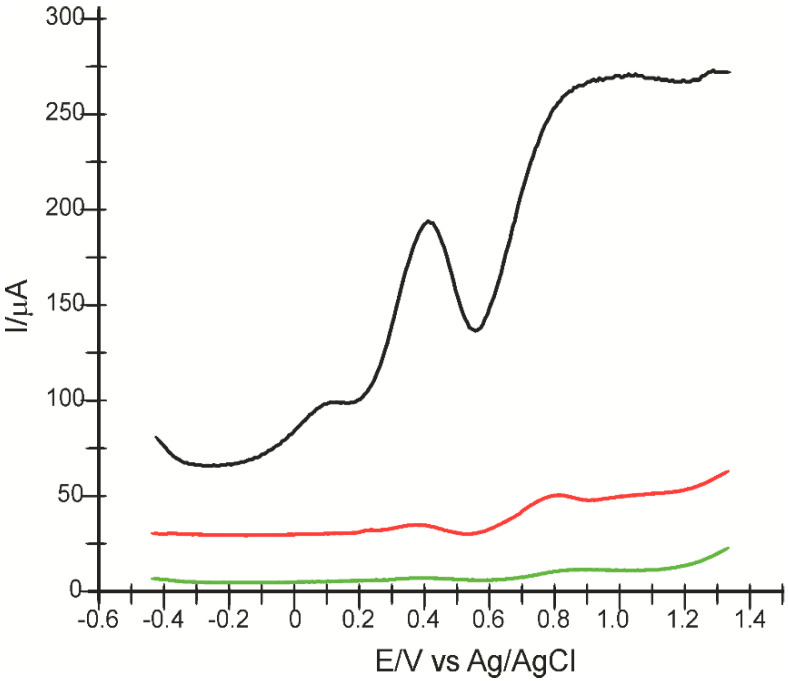
Square wave voltammograms obtained for CNF/SPE (black line), CNF-CoPc/SPE (red line), and CNF-CoPc-Lac/SPE (green line) by immersion in p-coumaric acid solution 10^−3^ M (PBS electrolyte 0.1 M pH = 5.0). The potential range is between −0.4 and +1.3 V, impulse height 0.09 V, potential increase 7 mV at a frequency of 15 Hz.

The same electrodes were used to record the square wave voltammograms in the p-coumaric acid solution 10^−3^ M (PBS electrolyte 0.1 M pH = 5.0). The potential range under study was between −0.4 and +1.3 V, the impulse height was 0.09 V, the increase in the impulse potential was 7 mV, and the frequency was 15 Hz. This technique yielded the same results as cyclic voltammetry. For all the three electrodes, two oxidation peaks stood out. In each situation, better defined peaks and lower background current were seen. By means of SWV, we were able to evince the reversibility of the peak corresponding to the oxidation product (P_ox_) and the irreversibility of the second peak (P_PCA_). For CNF-CoPc-Lac/SPE, the first anodic peak was observed at 0.392 V, and the second was observed at 0.885 V. Moreover, a much lower difference is remarked between the intensity of the first and the second anodic peak, which confirms that PCA is adsorbed on the sensor surface, interacting with the immobilized laccase. The square wave voltammograms are shown in Figure 7.

### 2.3. Influence of Scan Rate on the Voltammetric Response

**Figure 8:**
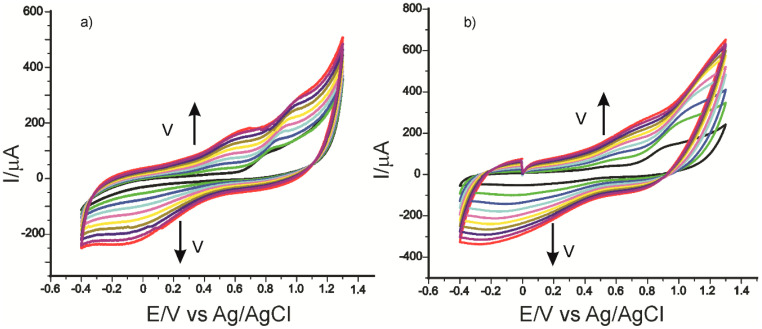
Cyclic voltammograms of CNF-CoPc/SPE (**a**) and CNF-CoPc-Lac/SPE (**b**) recorded at various scan rates within the range 0.1–1.0 V/s.

In the next stage, the electrochemical behavior of the three electrodes in PCA solution 10^−3^ M (the electrolyte support was 0.1 M PBS of pH 5.0) was studied, applying ever higher scan rates within the range 0.1–1.0 V/s. Significant differences are seen between the intensity of the oxidation and reduction currents and potentials measured, as early as the second scan rate applied, the peaks increasing progressively with the scan rate. Taking into consideration that enzyme immobilization predominantly influences the cathodic peak, the dependence of I_pc_ will be studied in relation to the scan rate. Figure 8 shows the cyclic voltammograms of CNF-CoPc-Lac/SPE recorded at various scanning rates.

It was found that there is a linear dependence between the currents of the cathodic peak and the scan rate for all the three electrodes (Table 2). It shows that the process occurring at the surface of the electrodes is controlled by the adsorption of the electroactive species, the adsorption of the hydroxycinnamic acid PCA on the active surface being the determining stage of the kinetics of the electrochemical process [63].

Taking into account the equation of the linear dependence between the cathodic peak current and the scan rate, the coverage degree of the electrode surface with the electroactive species (Γ) was calculated by means of the Laviron equation (1), and the results are shown in Table 2 [75].

(1) $$i_{\mathrm{pc}}=\frac{n^{2}F^{2}\Gamma \mathrm{Av}}{4\mathrm{RT}}$$

Comparing the results obtained with the three electrodes, it may be stated that in all the three cases, the reduction process is controlled by the PCA adsorption on the active surface, which is faster and more evident for the biosensor.

Table 2 shows the equation of the dependencies I_pc_ vs. v, the determination coefficients (R^2^), and the coverage degree of the electrode surface with the electroactive species (Γ).

The Γ values are in agreement with the ones obtained with other biosensors based on laccase, which are used in detecting phenolic compounds [76,77], and they have the greater value in the case of the biosensor.

These results clearly show that CNF-CoPc-Lac/SPE has higher electroanalytical properties for PCA detection. The laccase presence ensures biosensor selectivity, being used in testing complex samples. The immobilization of laccase together with carbon nanofibers and cobalt phthalocyanine leads to better bioselectivity and conductivity, these nanomaterials having a synergistic effect in biodetection [78].

Surface coverage concentration is close in value between the modified sensor and the biosensor. This could be the result of a thin layer of the enzyme or its low concentration. On the other hand, a thicker layer of the enzyme would have required a longer period for cross-linking with glutaraldehyde, which could affect the activity of the enzyme. In addition, cathodic currents of close intensities indicate that the enzyme could be degraded at higher scanning speeds.

However, since CNF-CoPc-Lac/SPE showed better sensitivity and selectivity performance, it will be used in further quantitative tests.

### 2.4. Calibration Curve

To develop the calibration curve, the cyclic voltammograms of p-coumaric acid were recorded, upon successive adding of variable amounts, between 5 and 30 μL, of stock solution of coumaric acid 10^−3^ M to 50 mL PBS pH 5.0 followed by stirring. After homogenizing the solution to be analyzed, the cyclic voltammograms were recorded. The concentration range was 0.1–202.5 μM.

**Figure 9:**
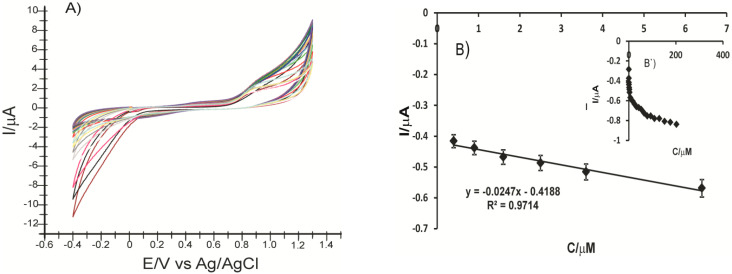
(**A**) Cyclic voltammograms recorded for CNF-CoPc-Lac/SPE on the concentration range 0.1–202.5 μM p-coumaric acid. Linear fitting within the range 0.4–6.4 μM (**B**) and 0.1–202.5 μM (**B**) for CNF-CoPc-Lac/SPE.

As it may be seen (Figure 9), the current of the cathodic peak increases with the increase in the PCA concentration. The current of the cathodic peak was linear within the range 0.4–6.4 μM.

By means of the linear regression equation, LOD (3σ/m, where σ was the standard deviation, and m the slope of the calibration curve) and LOQ (10σ/s) [79] were calculated, and the values may be seen in Table 3.

It may be seen that the biosensor is superior to the sensor in performance, due to the presence of the enzyme that provides selectivity and sensitivity and favors the interaction with p-coumaric acid.

**Figure 10:**
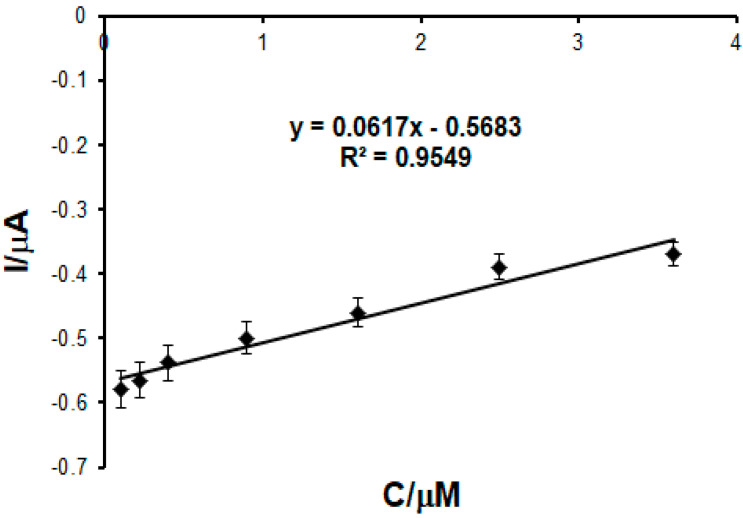
Linear fitting within the range 0.1–3.6 μM for CNF-CoPc-Lac/SPE; y = I (μA); x = c (μM); R^2^-coefficient of determination.

A calibration curve for the same concentration range (0.1–202.5 μM) of p-coumaric acid was also produced by chronoamperometry (Figure 10) for CNF-CoPc-Lac/SPE, when the potential was kept constant at −0.2 V. The LOD and LOQ values obtained were 1.63 × 10^−7^ and 5.42 × 10^−7^ respectively, which were close to those obtained by CV.

The low values of the detection and quantification limits are in agreement with the values obtained by other types of sensors or biosensors able to determine phenolic compounds, as seen in Table 4.

The biosensor proves high sensitivity, making a great difference due to the immobilization of laccase, which is an enzyme that also provides selectivity.

The voltamperometric methods proved feasible in the analysis of p-coumaric acid in various real-life examples, such as phytoproducts, which are useful in maintaining health or aiding in the treatment of certain conditions. To perform the quantitative analysis of p-coumaric acid in the selected phytoproducts, the new enzymatic study developed in this study may be used successfully.

### 2.5. Characteristics of the Biosensor

The storage stability of the developed biosensor was established in a continuous and an interrupted way. For this stage, two biosensors were prepared under the same conditions, and their sensitivity was studied by performing calibration curves for PCA over a week. While the response of the first biosensor was studied daily in triplicate during this time, the PCA signal of the second biosensor was assessed only on the first and last day of the study. The biosensors were stored at 4°C during the period when they were not used.

**Figure 11:**
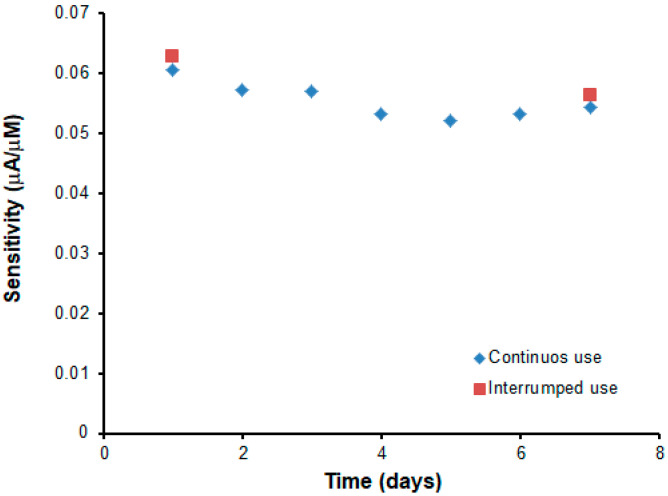
Evaluation of the storage stability of two PCA biosensors during one week by comparison of its sensitivity. When not in use, biosensors were stored at 4 °C.

The results in Figure 11 showed the evolution of sensitivity, demonstrating an excellent and similar storage stability in both cases, because the decrease was only 10.56 and 10.20% respectively.

In addition, operational stability was examined after 15 consecutive calibrations in one day. In this case, it was observed that the difference in sensitivity between the first and last calibration was reduced by 14%, which is an excellent value considering that SPEs are usually considered to be disposable.

### 2.6. Stability, Reproducibility of Fabrication, and Repeatability of the Biosensor

The stability of the biosensor was studied, and it was found that it may be used for more than 30 measurements by cyclic voltammetry in PCA solutions. To check the reproducibility of the manufacturing method, we studied the response of two identically prepared biosensors in PCA solutions of the same concentration. No differences higher than 2% were found between the two biosensors.

For a more detailed stability study, two other biosensors prepared in the same way were immersed in a 5 × 10^−3^ M PCA solution and two other were immersed in a 10^−2^ M PCA solution. The differences between the recorded currents between two biosensors were 1.8% and 1.9%, respectively.

Similarly, the variation of the biosensor’s response to PCA detection in a solution of the same concentration did not exceed 3% when taken out of the solution, rinsed, and subjected to the repetition of the cyclic voltammogram.

The repeatability of the measurements was investigated for five different biosensors. Biosensors were immersed one at a time in a 10^−3^ M p-coumaric acid solution (0.1 M PBS). The relative standard deviation of the measurements was 3.7%.

### 2.7. Interference Studies

For interference studies, the behavior of the biosensor was evaluated when adding compounds that are chemically related to PCA and are usually found in phytoproducts, such as for instance gallic acid, ascorbic acid, vanillic acid, and ferrulic acid. The PCA solution had a concentration of 50 μM, to which the same concentration of interfering substances was added. Subsequently, the interference concentrations increased, and the ratio between PCA and interference was 1:10, then 1:20.

The results may be seen in Table 5.

As it may be seen in Table 5, PCA determination is not significantly influenced by interfering compounds. The peaks pertaining to other compounds stand out, but the anodic peaks and the PCA cathodic peak are not influenced.

These results yield the conclusion that the CNF-CoPc-Lac/SPE sensor has good accuracy and selectivity for determining PCA in real-life samples.

### 2.8. Determining PCA in Phytoproducts

The selected phytoproducts for testing have various presentation forms: solid cream, cream, and capsules, with a composition rich in antioxidants. The manufacturer does not specify the exact PCA concentration in the prospectus, so that the quantitative determination by the voltammetric method will be validated by a traditional determination method. Well-established amounts of each product were used to obtain the solutions to be tested (Ghindazin 1 g, Tuiazin 1 g, Spirulin 0.75 g).

**Figure 12:**
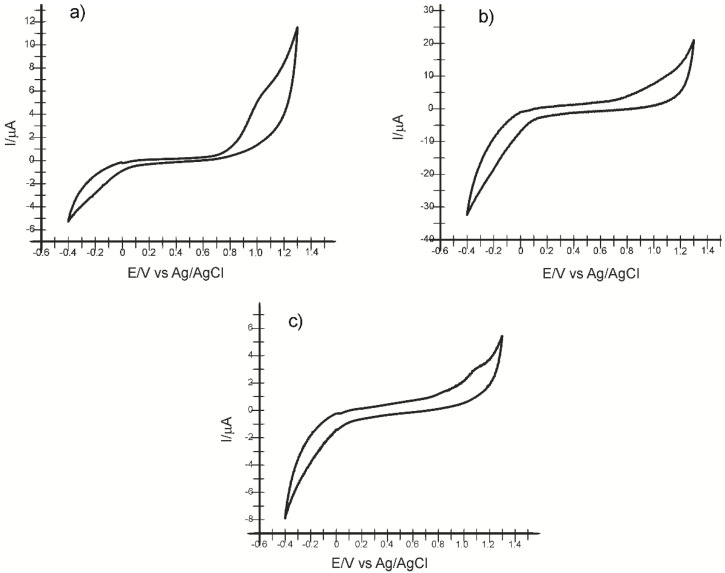
Cyclic voltammograms of the CNF-CoPc-Lac/SPE biosensor immersed in solution of (**a**) Spirulin, (**b**) Ghindazin, (**c**) Tuiazin, at 0.1 V/s scan rate.

Figure 12 shows the cyclic voltammograms of the CNF-CoPc-Lac/SPE immersed in solutions obtained from the products Spirulin, Ghindazin, and Tuiazin. The cyclic voltammograms recorded with CNF-CoPc-Lac/SPE exhibit the peaks related to the presence of p-coumaric acid in the samples tested.

The intensity of the cathodic peak related to the −0.2 V potential was used for quantification purposes in the case of each product. The results are shown in Table 4.

To validate the voltammetric method, the FTIR method was used. For the spectrometric analysis, five samples were prepared, with different concentrations of p-coumaric acid: 1, 2, 3, 4, and 5 mg/g, with KBr.

The samples were tested without preliminary preparation. The experiments were carried out in triplicate.

**Figure 13:**
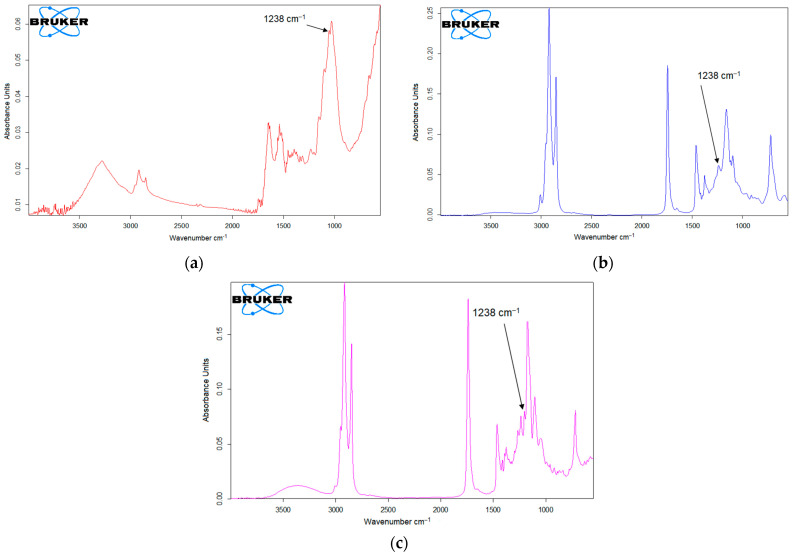
FTIR analysis of the products Spirulin (**a**), Ghindazin (**b**), and Tuiazin (**c**).

A calibration curve was drawn, according to the absorbance corresponding to the peak at 1238 cm^−1^, which is related to the elongation vibration of the C-O phenol group [31]. The calibration equation aided in calculating the amounts of p-coumaric acid in the phytoproducts. Figure 13 shows the spectra obtained for the commercial samples analyzed.

The values of the PCA concentration in the real samples calculated by the spectrometric method are close to the ones calculated by the voltammetric method, the data being shown in Table 6. These confirm the efficiency, sensitivity, and selectivity of the enzymatic sensor based on carbon nanofibers modified with cobalt phthalocyanine and laccase.

## 3. Materials and Methods

### 3.1. Reagents and Solution

To develop the biosensor, a screen-printed electrode based on carbon nanofibers (CNF/SPE) was purchased from Metrohm DropSens (Oviedo, Spain). CNF/SPE was subsequently modified in the lab. The modification with cobalt phthalocyanine (Fluka) was carried out by casting a 10^−5^ M CoPc solution in chloroform (Aldrich), obtaining CNF-CoPc/SPE. CNF-CoPc/SPE was modified in turn by immobilizing the laccase (Lac) enzyme followed by reticulation, thus reaching the CNF-CoPc-Lac/SPE biosensor.

Lac (from *Trametes versicolor* 0.78 U/mg) was purchased from Sigma-Aldrich, being used without supplementary purification. To immobilize the enzyme, a solution was used, which was obtained from laccase of 58.67 μg/µL concentration in PBS (0.1, pH 5.0).

To prepare the phosphate buffer solution (PBS 0.1 M) we used sodium diphosphate and phosphoric acid purchased from Sigma-Aldrich (Saint. Louis, Missouri USA). The amount of NaH_2_PO_4_ was calculated, weighed, and dissolved in ultrapure water obtained from a Milli-Q system (Millipore, Bedford, Massachusetts, USA). pH adjustment (pH = 3.0, 4.0, 5.0, 6.0) was performed by adding phosphoric acid, measuring the pH by means of a pH meter from WTW instruments, Weilheim, Germany.

The necessary amount of cobalt phthalocyanine (Fluka) was added to the chloroform to obtain a concentration of 10^−5^ M to be used in modifying the CNF/SPE electrode.

The p-coumaric acid was purchased from Sigma-Aldrich, being of analytical purity. The stock solution used had a 10^−3^ M concentration of p-coumaric acid, having as the support electrolyte the PBS solution, 0.1 M, pH 5.0.

The FTIR analysis used potassium bromide (Fluka) of analytic purity. The compounds used for the interference studies (ferrulic acid, vanillic acid, gallic acid) were purchased from Sigma-Aldrich. The L-ascorbic acid was bought from Riedel-de Haën (Seelze, Germania).

### 3.2. Electrodes and Devices Used

Electrochemical measurements were carried out by means of a conventional three-electrode system, a reference electrode Ag/AgCl (Princeton, Applied Research), an auxiliary electrode made of a platinum wire and a working electrode, which was CNF/SPE, CNF-CoPc/SPE, or CNF-CoPc-Lac/SPE.

The potentiostat/galvanostat used was EG&G, 263A model (Princeton Applied Research, Oak Ridge, TN, USA) controlled through Echem Software. The substances were weighed by the Partner AS 220/C/2 analytical scales and dissolved by means of an Elmasonic ultrasound bath (Carl Roth GmbH, Karlsruhe, Germany). The pH meter used to measure the pH was Inolab pH 7310, WTW instruments, Weilheim, Germany.

### 3.3. Preparation of the CNF-CoPc/SPE Biosensor

To prepare the modified CNF-CoPc/SPE biosensor, the following steps were taken: an amount of 10 μL cobalt phthalocyanine solution 10^−5^ M in chloroform was added on the surface of the screen-printed electrode modified with carbon nanofibers through the drop-and-dry technique, sequentially, with pauses to allow drying. Drying was performed at room temperature for 30 min. The addition of the cobalt phthalocyanine was carried out by an Eppendorf micropipette.

### 3.4. Preparation of the CNF-CoPc-Lac/SPE Biosensor

To prepare the biosensor, the support used was CNF-CoPc/SPE. A volume of 10 μL was added by the casting technique, sequentially, in two 3 h steps, with a drying pause between them. Enzyme reticulation was performed by placing the sensor above a 2 mL glutaraldehyde 2% container for 1 min.

**Figure 14:**
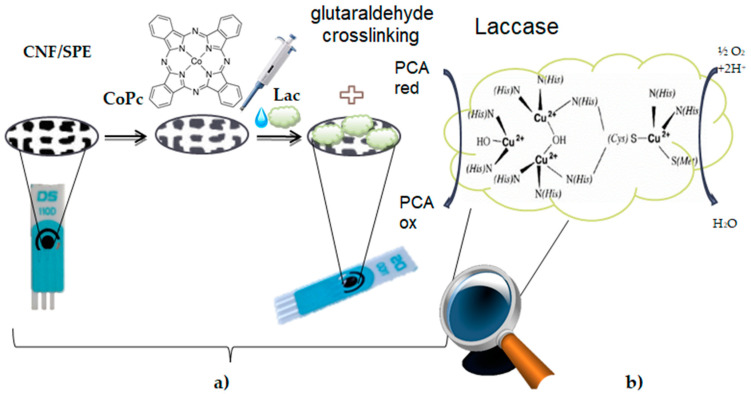
(**a**) Preparation process of the laccase-based biosensor on the support of a screen-printed electrode based on cobalt phthalocyanine-modified carbon nanofibers. (**b**) The enzymatic oxidation mechanism of PCA in the presence of laccase.

The glutaraldehyde vapors ensured the cross-linking of laccase on the electrode surface. The biosensors were stored at 4 °C for a maximum of 72 ore [85]. Figure 14 shows the preparation process of the biosensor (Figure 14a) and the enzymatic oxidation mechanism of PCA in the presence of laccase (Figure 14b).

### 3.5. Methods of Analysis

The voltammetric techniques are the most common stationary methods used in the analysis of phenolic compounds. The present study used three electroanalytical techniques to the purpose of validating results and better explaining the oxido-reduction processes occurring on the electrode surface.

#### 3.5.1. Cyclic Voltammetry

Cyclic voltammetry was used to characterize working electrodes as well as the stage of p-coumaric acid detection in the solution prepared with pure substance and in the solutions prepared with the real samples. The method is very suitable for these tests and provides valuable information on the electrochemical behavior of the substance under analysis [86,87]. The potential range was optimized, being between −0.4 and +1.3 V, and the scan rate varied from 0.1 to 1.0 V/s.

#### 3.5.2. Square Wave Voltammetry

Square wave voltammetry has a high testing rate and lower consumption of radioactive species, avoiding the damage to the electrode surface. The results obtained by the two voltammetric techniques were compared, providing further study opportunities. The great advantage of square wave voltammetry is the possibility to see during scanning if the electron transfer reaction is reversible or not. Since the current is measured during positive and negative impulses, the peaks corresponding to the oxidation or reduction of the radioactive species on the electrode surface may be obtained in the same experiment [46]. The potential range was from −0.4 to +1.3 V, the impulse height was 0.09 V, the frequency was 15 Hz, and there was an impulse potential increase of 7 mV.

#### 3.5.3. Chronoamperometry

Chronoamperometry was applied in the stage when the calibration curve was put together, and it provided information on the increase in the current intensity in relation to concentration, after applying the optimal potential to the working electrode. The applied potential was −0.2 V, and the set time was 900 s, as the volumes corresponding to the volumes in the stock solution were successively added at 1 min ranges.

### 3.6. Real-Life Samples and Preparation of Testing Solutions

The phytoproducts used for analysis were purchased from bio stores, upon considering the phytochemical composition and use directions. We selected three presentation forms: cream, solid cream, and tablet. All the three products have a diverse composition of active principles and excipients, and their prospectus states the presence of several hydroxycinnamic acids, including p-coumaric acid, the substance of interest in this study.

The cosmetic products chosen for testing were purchased from specialized stores based on their composition as indicated by the manufacturer. They had different presentation forms, i.e., serum, emulsion, and cream.

GHINDAZIN, solid cream (suppositories), is a patented product manufactured by Elzin Plant. Its composition is natural in origin, with extracts from various vegetable products: acorn (*Quercus glandem*), marigold (*Calendula Officinalis*), horse tail (*Equisetum arvense*), oak bark (*Quercus cortex*), propolis, oily extract of coniferous resin, beeswax (*Cera flava*), witepsol (palm oil 80% and coconut oil 20%).

Among the components of the products, there are essential oils, polysaccharides, terpenic acids, aromatic or aliphatic acids such as benzoic acid, phenyl-propanic derivatives (cinnamic, ferrulic, coumaric acid), resinols, glycoproteins, flavonoids, tannins, waxes, polysaccharides, polyphenolic acids, carotenoids, oligoelements, etc. Ghindazin is used for its antimicrobial, antibacterial, anti-inflammatory, antioxidant, analgesic, toning, detoxifying, hemostatic, and healing effect.

It is recommended as a support in case of hemorrhoids, anal fissures or fistulae, anal itching, rheumatism, muscle pain, and genital conditions (adnexitis, uterine fibroma, ovarian cysts).

The product is packaged as a box of blisters containing 10 suppositories (tronconic bars) of 1 g, 1.5 g, 2 g. Storage is only possible in the original package at a temperature of 15 °C. The product is EU-approved and may be purchased off the counter.

TUIAZIN (Elzin Plant) is a cream based on arborvitae, extracts of coniferous plants, and resins, and it is used as an adjuvant in treating various skin lesions, papillomas, or wounds that are difficult to heal.

The active principles recommending it are arborvitae (Tjuha), ivy (*Hedera helix*), basil (*Ocimum basilicum*), mallow (Malvae), horse tail (*Equisetum arvense*), tutsan (*Hypericum perforatum*), ribwort plantain (*Plantago lanceolata*), majoran (*Majorana hortensis)*, marigold (*Calendula Officinalis*), Swedish herbs, essential pine oil (*Pinus silvestris*), eucalyptus (*Eucalyptus globulus*), yellow bedstraw (*Galium verum*), oily extract of coniferous resin, beeswax (*Cera flava*), and distilled water.

The main components of the product are essential oils, polysaccharides, terpenic (resinic) acids, aromatic or aliphatic acids-benzoic acid, phenyl-propanic derivatives (cynamic, ferrulic, coumaric acid), resinols (higher aromatic alcohols, phenols, terpenoid alcohols), waxes, polysaccharides, glycoproteins, flavonoids, polyphenolic acids, etc. The product has an antiseptic, analgesic, toning, anti-edema, anti-inflammatory, comforting, healing, antirheumatic, anticargenogenic effect, etc.

SPIRULIN AND BUCKTHORN (Dacia Plant) is a natural product recommended for the body’s remineralization and vitamin supply, helping it improve its resilience and general well-being. Spirulin is very rich in proteins, B complex vitamins (B_1_, B_2_, B_6_, folic acid), beta-carotene, vitamin E, mineral salts (iron, calcium, zinc, potassium, magnesium, selenium, copper, and sodium), fatty acids, phycocyanine, chlorophyll, etc.

In dried form, 100 g of spirulin contain 60–70 g protein, being easily absorbed by the human body. The buckthorn fruit is an important source of vitamin C. The plant also contains vitamins B_1_, B_2_, E, P, PP, provitamins D, carotenoides (α si β-carotene, lycopene, zeaxanthin), folic acid, flavonoids (syringetin, isorhamnetin, quercetin, kaempferol, myricetin), proantocyans, phenollic acids (gallic, vanillic, salicylic, cynamic, protocatechic, caffeic, ferrulic, p-coumaric), trans-resveratrol, triterpenic acids (ursolic and oleanolic acid), lipids (glycerides of the linoleic, linolenic, palmytic, oleic acids,), oligoelements (Ca, P, Mg, K, Fe, Na), sterols (β-sitosterol), sugars, etc.

Different amounts of each product were used for the electrochemical analysis, according to the presentation form and the possibility of obtaining fractions.

From the Ghindazin product, a suppository (1 g) was sampled, which was subsequently melted in a water bath together with 50 mL PBS solution 0.1 M, pH 5.0, and ultrasonicated for homogenization.

From the Tuiazin product, 1 g was weighed on the analytical scales; then, it was dispersed and homogenized in the same solvent.

The capsule of Spirulin and Buckthorn (0.75 g) was triturated in the mortar and dispersed in PBS 0.1 M pH 5.0. Each sample was filtered through filter paper and analyzed separately (3 replications for each sample).

The voltammetric techniques used were cyclic voltammetry and square wave voltammetry in the potential range between −0.4 and 1.3 V. To validate the voltammetric method, the FTIR spectra were recorded for the pure PCA substance of various concentrations 1, 2, 3, 4, and 5 mg/g and the phytoproducts samples, by means of a FTIR spectrophotometer Bruker ALPHA (BrukerOptik GmbH, Ettlingen, Germany), in the 4000–500 cm^−1^ range in ATR mode. The ZnSe crystal of the device was cleaned with isopropanol after each sample in order to remove any impurities that might influence the results.

## 4. Conclusions

The present study proved the feasibility of developing modified sensors: one with cobalt phtalocyanine and the other with cobalt phtalocyanine and laccase, which are used to determine p-coumaric acid in phytopreparations. The results obtained prompted the conclusion that cobalt phtalocyanine favored the activity of laccase, also being a mediator of electron transfer in the oxidation process of p-coumaric acid.

The voltamperometric methods used in biodetection were cyclic voltammetry and square wave voltammetry. Cyclic voltammetry and chronoamperometry were used to study the electrochemical behavior of the biosensor within the selected concentration range.

The enzymatic biosensor shows high sensitivity and selectivity in the amperometric detection of hydroxycinnamic acid. The LOD and LOQ values obtained by CNF-CoPc-Lac/SPE are close to those obtained by other laccase-based biosensors when detecting phenolic compounds.

The PCA concentrations obtained by CNF-CoPc-Lac/SPE were close to those obtained by the FTIR spectrometric method.

In conclusion, the new biosensor developed based on cobalt phtalocyanine and laccase has multiple advantages, such as sensitivity, selectivity, reliability, and low cost. In addition, CNF-CoPc-Lac/SPE may be used in routine tests for the control of nutraceuticals, food, and pharmaceutical products.

## Figures and Tables

**Table 1:** The values of the parameters obtained from the cyclic voltammograms of all the electrodes immersed in 10^−3^ M p-coumaric acid solution (the electrolyte support was 0.1 M PBS of pH 5.0).

Electrode	E_pa_^1^ (V)	E_pox_^2^ (V)	E_pc_^3^ (V)	E_1/2_^4^ (V)	I_pa_^5^ (µA)	I_pox_^6^ (µA)	I_pc_^7^ (µA)	I_pc_/I_pox_
CNF/SPE	0.814	0.523	0.160	0.341	171.170	30.300	−20.219	0.669
CNF-CoPc/SPE	0.818	0.489	0.079	0.284	96.208	19.650	−25.354	1.290
CNF-CoPc-Lac/SPE	0.904	0.537	0.011	0.274	147.964	40.269	−52.506	1.303

E_1/2_ = (E_pox_+ E_pc_)/2.

**Table 2:** The linear fitting equations (I_pc_ vs. v), R^2^, and Γ for the three electrodes used in the analysis.

Electrode	Equation	R^2^	Γ (mol × cm^−2^)
CNF/SPE	I_pc_ = −197.46 × 10^−6^ v −7.5975 × 10^−6^	0.996	3.84 × 10^−10^
CNF-CoPc/SPE	I_pc_ = −229.75 × 10^−6^ v −11.029 × 10^−6^	0.999	4.46 × 10^−10^
CNF-CoPc-Lac/SPE	I_pc_ = −310.01 × 10^−6^ v −41.585 × 10^−6^	0.982	6.02 × 10^−10^

**Table 3:** Equation of linear dependence between I_pc_ and c, R^2^, LOD, and LOQ for CNF-CoPc-Lac/SPE.

Electrode	Linear Equation	R^2^	LOD (M)	LOQ (M)
CNF-CoPc-Lac/SPE	I_pc_ = −0.0247c − 0.4188	0.9714	4.83 × 10^−7^	1.61 × 10^−6^

**Table 4:** Phenolic compounds, detection technique, linearity range, and LOD of some laccase-based biosensors.

Laccase Biosensor	Analyte	Detection Technique	Linearity Range (µM)	LOD (M)	Ref.
Lac/Ag-ZnONPs/MWCNTs/C-SPE	Bisphenol A	CV	0.5–2.99	6.0×10^−9^	[42]
ePDA-Lac	Caffeic acid	CV	1–50	1.4 × 10^−7^	[80]
	Rosmarinic acid		1–20	9.0 × 10^−8^	
	Gallic acid		1–150	2.9 × 10^−7^	
Lac/MWCNT-COOH/AuNPs-SDBS-PEDOT/GCE	Catechol	DPV	0.1–0.5 11.99–94.11	1.1 × 10^−7^ 1.22 × 10^−5^	[81]
Nafion-TiO_2_/CuCNFs-Lac-GCE	Hydroquinone	Amp	1–89.8	3.65 × 10^−6^	[82]
FYSSns–2–Lac/GCE	Catechol	DPV	12.5–450	1.6 × 10^−6^	[83]
PtNPs-BOT-Lac/GO	5-CQA	SWV	0.56–7.3	1.8 × 10^−7^	[41]
GNP@MnO_2_ (SPCE/GNP@MnO_2_)	caffeic acid	Amp	-	1.9 × 10^−6^	[84]
CNF-CoPc-Lac/SPE	p-coumaric acid	CV	0.4–6.4	4.83 × 10^−7^	This work

Lac/Ag-ZnONPs/MWCNTs/C-SPE carbon screen-printed electrode modified with multiwalled carbon nanotubes that are functionalized with silver-doped zinc oxide nanoparticles (Ag-ZnONPs) on which the laccase enzyme was immobilized. ePDA-Lac -laccase-polydopamine sensor. Lac/MWCNT-COOH/AuNPs-SDBS-PEDOT/GCE laccase on a glassy carbon electrode modified by conducting polymers built of poly(3,4-ethylenedioxythiophene), gold nanoparticles, and carboxylated multiwalled carbon nanotubes. Nafion-TiO_2_/CuCNFs-Lac-GCE biosensor was prepared on the basis of laccase, Nafion, and TiO_2_-loaded copper and carbon composite nanofibers (TiO_2_/CuCNFs). FYSSns-2-Lac/GCE laccase biosensor manufactured from flower-shaped yolk-shell SiO_2_ nanospheres. GNP@MnO_2_ (SPCE/GNP@MnO_2_) laccase polyphenolic biosensor supported on graphene nanoplatelets (GNP) and manganese(IV)-oxide (MnO_2_) nanoparticles-decorated carbon screen-printed electrode.

**Table 5:** Interference of chemically related compounds with the quantitative determination of PCA 10^−5^ M.

Interfering Compound	Ratio	Recovery/%	Ratio	Recovery/%	Ratio	Recovery/%
ascorbic acid	1:1	101 ± 3.6	1:10	99 ± 2.6	1:20	98 ± 2.8
ferrulic acid	1:1	99 ± 2.7	1:10	98 ± 3.1	1:20	97 ± 3.3
vanillic acid	1:1	98 ± 3.9	1:10	96 ± 4.0	1:20	95 ± 3.6
gallic acid	1:1	102 ± 3.7	1:10	100 ± 2.2	1:20	99 ± 3.2

**Table 6:** PCA concentrations in phytoproducts obtained by the voltammetric method and the FTIR method, respectively.

Phytopreparations	FTIR Method mg/g PCA	Voltammetric Method mg/g PCA
Spirulin	1.569	1.674
Ghindazin	0.644	0.783
Tuiazin	1.936	2.149

## Data Availability

The authors confirm that the data supporting the findings of this study are available within the article.
